# A phase Ib/II study of galunisertib in combination with nivolumab in solid tumors and non-small cell lung cancer

**DOI:** 10.1186/s12885-023-11153-1

**Published:** 2023-07-28

**Authors:** Ernest Nadal, Mansoor Saleh, Santiago Ponce Aix, Maria Ochoa-de-Olza, Sandip Pravin Patel, Scott Antonia, Yumin Zhao, Ivelina Gueorguieva, Michael Man, Shawn T. Estrem, Jiangang Liu, Emin Avsar, Wen Hong Lin, Karim A. Benhadji, Leena Gandhi, Susan C. Guba, Inmaculada Ales Diaz

**Affiliations:** 1 Department of Medical Oncology, Catalan Institute of Oncology, IDIBELL, L’Hospitalet, Barcelona, Spain; 2 University of Alabama, Birmingham, AL USA; 3 Hospital 12 de Octubre – Oncology, Madrid, Spain; 4 Hospital Universitario Vall d’Hebron, Barcelona, Spain; 5 University of California, San Diego, CA USA; 6 H. Lee Moffitt Cancer Center and Research Institute, Tampa, FL USA; 7 Eli Lilly and Company, Indianapolis, IN USA; 8 Bristol Myers Squibb, Princeton, NJ USA; 9 Immune-Onc Therapeutics, Palo Alto, CA USA; 10 Dana-Farber Cancer Institute, Harvard Medical School, Boston, MA USA; 11 UGCI Oncología Médica, Hospitales Universitarios Regional Y Virgen de La Victoria, IBIMA, Málaga, Spain

**Keywords:** Galunisertib, Immune checkpoint inhibitor, Nivolumab, NSCLC, TGF-β

## Abstract

**Background:**

In this phase Ib/II open-label study, tumor immune suppression was targeted in patients with advanced refractory solid tumors and patients with recurrent/refractory non-small cell lung cancer (NSCLC) using galunisertib with nivolumab.

**Methods:**

Eligible patients were ≥ 18 years old, had an Eastern Cooperative Oncology Group performance status ≤ 1, and were treatment-naive for anti-programmed cell death-1, its ligand, or transforming growth factor β receptor 1 kinase inhibitors. Phase Ib was an open-label, dose-escalation assessment of the safety and tolerability of galunisertib with nivolumab in patients with advanced refractory solid tumors. Phase II evaluated the safety of galunisertib with nivolumab in NSCLC patients who had received prior platinum-based treatment but were immuno-oncology agent-naive.

**Results:**

This trial was conducted between October 2015 and August 2020. No dose-limiting toxicities were observed in phase I. In the phase II NSCLC cohort (*n* = 25), patients received 150 mg twice daily galunisertib (14 days on/14 days off dosing schedule for all phases) plus nivolumab at 3 mg/kg (intravenously every 2 weeks). In this phase, the most frequent treatment-related adverse events (AEs) were pruritus (*n* = 9, 36%), fatigue (*n* = 8, 32%), and decreased appetite (*n* = 7, 28%). No grade 4 or 5 treatment-related AEs were observed. Six (24%) patients had confirmed partial response (PR) and 4 (16%) had stable disease; 1 additional patient had confirmed PR after initial pseudo-progression. The median duration of response was 7.43 months (95% confidence interval [CI]: 3.75, NR). Among the 7 responders, including the delayed responder, 1 had high PD-L1 expression (≥ 50%). The median progression-free survival was 5.26 months (95% CI: 1.77, 9.20) and the median overall survival was 11.99 months (95% CI: 8.15, NR). Interferon gamma response genes were induced post-treatment and cell adhesion genes were repressed, although the association of these observations with tumor response and clinical outcomes was not statistically powered due to limited samples available.

**Conclusions:**

The study met its primary endpoint as galunisertib combined with nivolumab was well tolerated. Preliminary efficacy was observed in a subset of patients in the Phase 2 NSCLC cohort.

**Trial registration:**

Trial registered with ClinicalTrials.gov (NCT02423343; 22.04.2015).

**Supplementary Information:**

The online version contains supplementary material available at 10.1186/s12885-023-11153-1.

## Background

Non-small cell lung cancer (NSCLC) accounts for approximately 85% of the incidences of lung cancer [1, 2]. Many tumors, including NSCLC, progress in part due to the acquisition of traits that allow cancer cells to evade immunosurveillance and escape the immune response.

Cancer immunotherapy aims to activate the host’s immune system to recognize and attack cancer cells. Recent successes in immunotherapy have been achieved by targeting immune checkpoints, i.e. a host of inhibitory receptors expressed on immune cells that upon activation suppress the inflammatory response [3]. Immune checkpoint inhibitors can induce durable responses and have demonstrated a survival benefit in patients with cancer, including metastatic, locally advanced, and early stage NSCLC [4–7].

Programmed cell-death-1 (PD-1) is expressed on activated T-cells and can act to dampen the immune response [8]. Tumor cells overexpress PD-1 ligand (PD-L1), either by pro-inflammatory stimuli or as a result of pro-oncogenic pathway activation, and inhibit the local immune response [9]. Nivolumab blocks the binding of PD-L1 and PD-L2 to its receptor, allowing the activated T-cells to identify and attack cancer cells [10]. Although NSCLC may have a high mutational load or PD-L1 expression, which are associated with response to anti-PD(L)1 therapy, many patients do not respond to single agent anti-PD(L)1 inhibition [11, 12].

Transforming growth factor β (TGF-β) signaling plays an important role in tumorigenesis and contributes to many hallmarks of cancer cells including cell proliferation, invasion, escape of immune surveillance, angiogenesis, and metastasis [13]. A major contributing factor for mortality in NSCLC, like most cancer types, is metastasis [14]. Epithelial-mesenchymal transition (EMT) is a crucial event leading to metastasis during which cells exhibit mesenchymal properties such as becoming more motile and invasive [15]. TGF-β signaling is a primary inducer of EMT in NSCLC [16]. TGF-β binding to its receptor leads to phosphorylation of small mothers against decapentaplegic homolog 2 (SMAD2) and SMAD3, which then form a transcriptional complex with SMAD4, activating the expression of target genes [17]. SMAD2 has been identified as a key element downstream of the TGF-β signaling pathway in regulating cancer metastasis through promoting EMT [18, 19]. Upregulation of SMAD2 has been associated with poor survival in patients with NSCLC [20].

Galunisertib is an oral small molecule inhibitor of the TGF-β receptor 1 kinase that specifically down-regulates the phosphorylation of SMAD2 and is associated with an increase in T-cell infiltration in tumors [21, 22].

TGF-β can suppress or alter the activation, maturation, and differentiation of both innate and adaptive immune cells [23]. Increased TGF-β in the tumor microenvironment promotes T-cell exclusion from tumors, and blocks acquisition of the T helper cells-effector phenotype, which are both associated with poor clinical outcomes. Inhibition of TGF-β releases a cytotoxic T-cell response against tumor cells and allows immune cells to infiltrate the tumor [24]. However, inhibition of TGF-β on its own is not always sufficient to promote tumor rejection [23]. In preclinical models, concurrent blockade of TGF-β and PD-L1 work synergistically to reverse immunosuppression leading to T-cell infiltration and activation, which promote antitumor activity [24–26].

In this study, both TGF-β and PD-1 were targeted using galunisertib in combination with nivolumab in patients with advanced refractory solid tumors and in patients with recurrent/refractory NSCLC who were PD-(L)1 naïve. The clinical trial objectives were to evaluate the safety and tolerability of the drug combination.

## Patients and methods

### Study design

This was a phase Ib/II open-label study (NCT02423343) conducted between October 2015 and August 2020. The phase Ib portion of this study consisted of a dose-escalation assessment of the safety and tolerability of galunisertib administered at 50 mg daily, 50 mg twice daily (BID), 80 mg BID, or 150 mg BID in combination with nivolumab 3 mg/kg administered every two weeks in patients with advanced refractory solid tumors. The phase II portion of the trial evaluated the safety and efficacy of 150 mg BID galunisertib in combination with nivolumab at 3 mg/kg in patients with recurrent or refractory NSCLC that were immune checkpoint naive. The study did not have a fixed treatment duration, and patients received combination treatment in 28-day cycles until disease progression, intolerable toxicity or withdrawal of consent. Based on the results of a preclinical dosing schedule investigation that evaluated toxicity using animal models [27], each patient took galunisertib on an intermittent dosing strategy of a 14 days on/14 days off dosing schedule for all phases of the trial. 

### Eligibility criteria

Inclusion and exclusion criteria for phase Ib and phase II are shown in Additional file 1. In the phase 1a escalation portion, patients with any solid tumor in the advanced stage refractory to standard of treatment were eligible. The phase II portion of this study included patients with stage IV NSCLC who were previously treated with platinum-based chemotherapy. Patients with actionable oncogene mutations were required to receive prior treatment with tyrosine kinase inhibitor (TKI) (e.g., approved EGFR TKI for patients with activating EGFR mutations and approved ALK TKI for patients harboring ALK-fusions). Patients that received more than 1 line of treatment in the advanced setting were excluded. Eligible patients were ≥ 18 years old with an Eastern Cooperative Oncology Group (ECOG) performance status ≤ 1, were treatment-naive for anti-PD-(L)1 or TGF-β receptor 1 kinase inhibitors and had measurable disease per Response Evaluation Criteria in Solid Tumors (RECIST) v1.1 [28].

### Efficacy measures

Efficacy measures included overall survival (OS), progression-free survival (PFS), overall response rate [ORR -partial response (PR) + complete response (CR)] and duration of response (DoR). Tumor responses were assessed per RECIST v1.1 guidelines. DoR, PFS and OS were estimated using Kaplan- Meier methodology.

### Outcomes

The primary objective of the study (both phases) was to assess the safety and tolerability of galunisertib in combination with nivolumab by identifying dose-limiting toxicities (DLTs) and the maximum tolerated dose or pharmacologically active dose of the combination in patients with advanced refractory or solid tumors during the first 2 cycles. DLTs were defined as Grade 3 non-haematologic toxicity; Grade 4 haematological toxicity of > 5 days duration; any febrile neutropenia.

The secondary objectives of the study were to characterize the pharmacokinetics (PK) of galunisertib and nivolumab when co-administered, to characterize the immunogenicity of nivolumab when administered in combination with galunisertib, and to estimate the OS rate. In addition, PFS, ORR, and DOR were evaluated for patients with NSCLC in phase II. Exploratory objectives were to examine biomarkers and correlate these makers to clinical outcomes.

### Toxicity and safety measures

All patients who received at least 1 dose of either galunisertib or nivolumab were evaluated for safety and toxicity. Any other significant toxicity deemed to be dose limiting or resulted in the patient getting < 75% of the total doses or lead to holding galunisertib for > 2 weeks. For nivolumab, any toxicity that occurred during Cycles 1 or 2 managed by discontinuation. Any Grade ≥ 2 nonskin, drug-related AE; any Grade 3 skin, drug related AE; any Grade 3 drug-related laboratory abnormality; any adverse event (AE), laboratory abnormality, or intercurrent illness which, in the judgment of the investigator, warranted delaying the dose of study medication. Exceptions not considered a DLT included: Grade 3 amylase or lipase abnormalities not associated with symptoms or clinical manifestations of pancreatitis that did not require a dose delay; nausea, vomiting, diarrhea, and constipation controlled with treatment; fatigue relieved by rest; Grade 3 elevations of alanine aminotransferase and/or aspartate aminotransferase, without evidence of other hepatic injuries; anorexia not associated with significant weight loss (weight loss of > 10% baseline) or malnutrition. The AEs were assessed, according to the Common Terminology Criteria for Adverse Events (CTCAE) version 4.0, continuously during the study and for 100 days after the last dose of combination treatment. Disease assessment with computed tomography and/or magnetic resonance imaging, as appropriate, were performed at baseline and approximately between Days 22 and 28 of every other cycle and completed before the first dose in the next cycle, until disease progression or patient withdrawal from the study.

### Pharmacokinetics and biomarkers

PK of galunisertib were measured using validated liquid chromatography-atmospheric pressure ionization/tandem mass spectrometry methods at Intertek Pharmaceutical Services (El Dorado Hills and San Diego, California, USA). There were 293 PK galunisertib observations in 41 patients on Days 1, 14, and 15 in Cycles 1 and 2 as well as pre-dose, Cycle 4 Day 1 (C1D4).

Tumor cell membrane PD-L1 was measured by immunohistochemistry using the Dako PD-L1 (28–8) kit at Mosaic Laboratories (*n* = 20 samples) and scored as percent tumor cells expressing PD-L1. Anti-drug antibodies (ADA) were evaluated in all patients comparing their baseline levels to study levels. Patients that developed positive ADA after negative baseline were further evaluated for AEs and response to treatment to assess potential relationship between study treatment and immunogenicity.

Cancer gene sequencing (*n* = 9 tumor samples) was performed by Foundation Medicine using the FoundationOne T7 assay (404 genes). Here we report results for tumor mutation burden (TMB) as mutations/megabase and genetic variants for common mutated genes or genes with known relevance to anti-PD(L)1 NSCLC response.

Ribonucleic acid (RNA) extraction and Illumina TruSeq RNA Exome sequencing was performed by Almac for gene expression profiling of 12 baseline tumor samples, 4 tumors collected on C1D15, and 3 tumors collected on C2D1. Six baseline samples were paired with treatment biopsies. Formalin fixed paraffin embedded tumor samples were macrodissected and RNA extraction was performed using the Qiagen RNeasy extraction kit followed by quality assessments using Nanodrop, Agilent Bioanalyzer & RNA-Seqability (an Almac proprietary quality control step). Paired-end sequencing with a read length of 100 base pairs and targeted read depth of 50 million reads/sample was performed. Read counts were quantified using an in-house Perl script and summarized at the gene level (National Center for Biotechnology Information [NCBI] h37.p13 annotation). The resulting data were quantile-normalized and filtered to remove genes with fewer than 5 counts across 80% of the samples from the analysis. Baseline expression of TGF-β pathway genes included *TGFβ-1, TGFβ-2, ACVR1, TGFβ-3, TGF-β R1, TGF-β R2, SMAD2, SMAD4*; and T-cell inflamed genes included *IFNGR1, IFNG, TIGIT, CD27, CD8A, PDCD1LG2, LAG3, CD274, CXCR6, CMKLR1, NKG7, CCL5, PSMB10, IDO1, CXCL9, HLA-DQA1, CD276, STAT1, HLA-DRB1*, and *HLA-E*.

Differentially expressed gene analysis was conducted using the DESeq2 package [29]. Fold changes were calculated to show up or down-regulation of genes between 4 tumors collected on C1D15 and 3 tumors collected on C2D1 versus 12 baseline tumor samples. Differentially expressed genes were identified from comparisons when the *p*-value was ≤ 0.05 and the absolute fold change was ≥ 1.5. The contrasts of differentially expressed genes were displayed in a heatmap using the Complex Heatmap R package (https://bioconductor.org/packages/release/bioc/html/ComplexHeatmap.html).

Serum proteins (51 analytes) were assessed at baseline, C1D8, C1D15, C2D1, and C2D15 using the Inflammation Multi-Analyte Immunoassay panel developed by Myriad RBM. Paired t-tests were used to compare post-dose levels with baseline at each time point for each biomarker.

### Statistical analysis

All patients who received any dose of study treatment were included in safety and efficacy analyses. For safety analyses, the frequency and percentage of patients with dose-limiting toxicities and AEs were presented for each cohort. Best overall response per RECIST v1.1 with confirmation on CR and PR were represented by frequency, percentage, and 95% confidence interval (CI) with the Clopper-Pearson Confidence Interval method. PFS, DOR, and OS were analyzed using the Kaplan–Meier method. PFS was defined as the time from the date of first study treatment to the first date of documented progression or death due to any cause. For patients who were not known to have died or progressed as of the cut-off dates, PFS times were censored at the date of the last progression-free disease assessment prior to the date of any subsequent anticancer therapy. DOR was measured from the date of the first documented response to the date of first disease progression or the date of death due to any cause, using the same censoring rules as PFS. OS was defined as the time from the date of first study treatment to the date of death from any cause. For each patient who was not known to have died as of the cut-off date, OS data were censored for that analysis at the date of last contact prior to the data inclusion cutoff date. SAS 9.4 was used for analyses on the clinical data for demographics, baseline characteristics, safety, and efficacy.

## Results

### Baseline patient demographics

Fifteen patients were enrolled in phase Ib and 25 patients in phase II. Baseline patient and disease characteristics of patients in phase 1b are depicted in Additional file 2. In the phase II NSCLC cohort, 16 (65%) of the patients were male and all were Caucasian. Two (8%) had tumors with PD-L1 expression of  ≥ 50%, 11 (44%) had PD-L1 expression ranging from 1–49%, and 7 (28%) had no PD-L1 expression. Demographic and baseline disease characteristics of the patient population are shown in Table 1.

**Table 1:** Phase II baseline patient demographics

Characteristics	Phase II NSCLC Cohort (*n* = 25) n (%)
Sex	
Male	16 (64)
Age, median (range)	61 (43–80)
Race	
White	25 (100)
Ethnicity	
Hispanic or Latino	5 (20)
Not Hispanic or Latino	18 (72)
Unknown	2 (8)
Baseline ECOG performance status	
0	3 (12)
1	22 (88)
Tobacco use	
Current	6 (24)
Former	17 (68)
Never	2 (8)
Prior therapy	
1 prior regimen	23 (92)
2 prior regimens ^a^	2 (8)
PD-L1 status	
≥ 50%	2 (8)
1–49%	11 (44)
< 1%	7 (28)
Data not available	5 (20)

*Abbreviations*: *ECOG* Eastern Cooperative Oncology Group, *n* number of patients, *NSCLC* non-small cell lung cancer, *PD-L1* programmed death ligand 1

^a^ One patient received adjuvant chemotherapy and another patient received neoadjuvant chemotherapy

### Toxicity and safety

The primary objective of safety was reached for both phases of the trial. No DLTs were observed in the phase Ib portion of the study. The median duration of galunisertib treatment ranged from 56 days (min 28 days; max 157 days) to 143 days (min 55 days; max 462 days) across the phase Ib cohorts. The most common AEs related to study treatment in phase Ib were observed in the galunisertib 150 mg BID cohort and included rash maculo-papular, amylase increased, aspartate aminotransferase increased, gamma-glutamyltransferase increased, blood alkaline phosphatase increased, lipase increased, and non-cardiac chest pain. The median duration of galunisertib treatment was 120 days (min 14 days; max 726 days) in the phase II NSCLC cohort. Two deaths occurred on treatment (multi-organ failure and myocardial infarction) in phase II. Both cases were deemed unrelated to study treatment by the treating physicians. The case of multi-organ failure was deemed related to study disease and, the case of myocardial infarction was considered unrelated as patient had pre-existing underlying conditions that increased the risk of cardio-vascular disease, and a familial history of cardiac disease. There were no Grade 4 or 5 treatment-related toxicities. The most frequent treatment-related AEs were pruritus (*n* = 8, 32%), fatigue (*n* = 8, 32%), and decreased appetite (*n* = 7, 28%). Other treatment-related Grade 3 AEs included immune-related encephalitis, diarrhea, fatigue, aspartate aminotransferase/alanine aminotransferase/gamma-glutamyltransferase increase, blood alkaline phosphatase increase, abdominal distension, cutaneous rash (*n* = 1 each), and cholestasis (*n* = 2) that resolved or were resolving at the time of data cutoff (Table 2). A total of 28 patients (68%) across the phase Ib cohorts and the phase II NSCLC cohort experienced a Grade ≥ 3 AE, of which 12 (29%) were related to treatment. Discontinuation rate due to any treatment related AE was 2%.

**Table 2:** Treatment-emergent adverse events related to study treatment per investigator assessment

Treatment-Emergent Adverse Events Related to Study Treatment (> 10% and Grade 3)	Phase II NSCLC Cohort (*n* = 25) n (%)	
	**Any Grade**	**Grade 3**^a^
Pruritus	8 (32)	–
Fatigue	8 (32)	1 (4)
Decreased appetite	7 (28)	–
Diarrhea	5 (20)	1 (4)
Maculo-papular rash	5 (20)	–
Nausea	4 (16)	–
Dry mouth	4 (16)	–
Hypothyroidism	3 (12)	–
Cholestasis	3 (12)	2 (8)
Abdominal distension	3 (12)	1 (4)
Aspartate aminotransferase increased	2 (8)	1 (4)
Alanine aminotransferase increased	2 (8)	1 (4)
Gamma-glutamyltransferase increased	2 (8)	1 (4)
Blood alkaline phosphatase increase	1 (4)	1 (4)
Encephalopathy	1 (4)	1 (4)

*Abbreviations*: *n* number of patients *NSCLC* non-small cell lung cancer

^a^ No Grade 4 or 5 treatment-related toxicity

### Efficacy

**Fig. 1:**
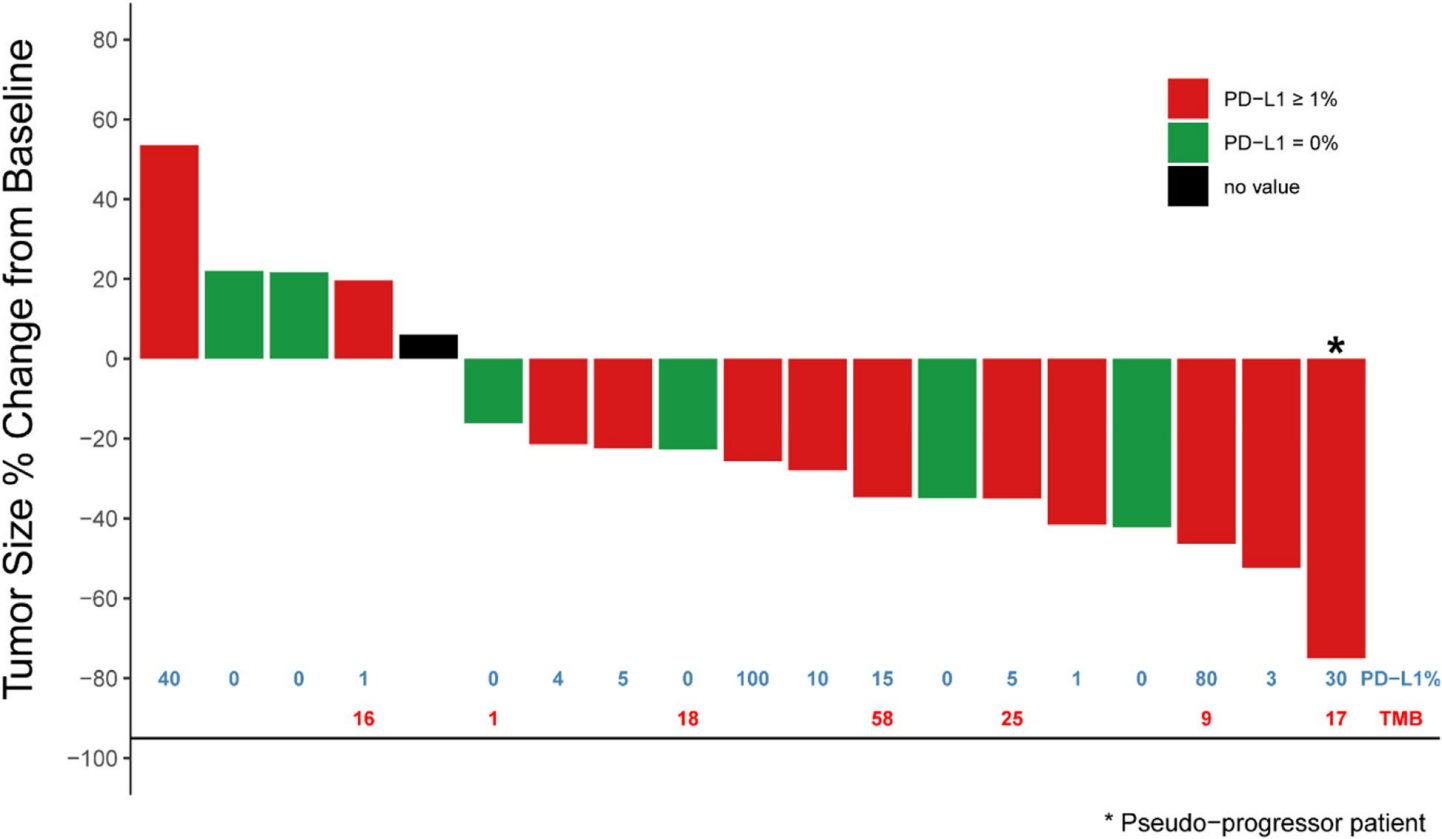
Waterfall plot of best overall response rate (ORR) according to RECIST in the in NSCLC Cohort. Information is provided about baseline PD-L1 expression in 18 patients (number in blue), and tumor mutation burden (TMB) in 7 patients (number in red). Abbreviations: NSCLC, non-small cell lung cancer; PD-L1, programmed death ligand 1

**Fig. 2:**
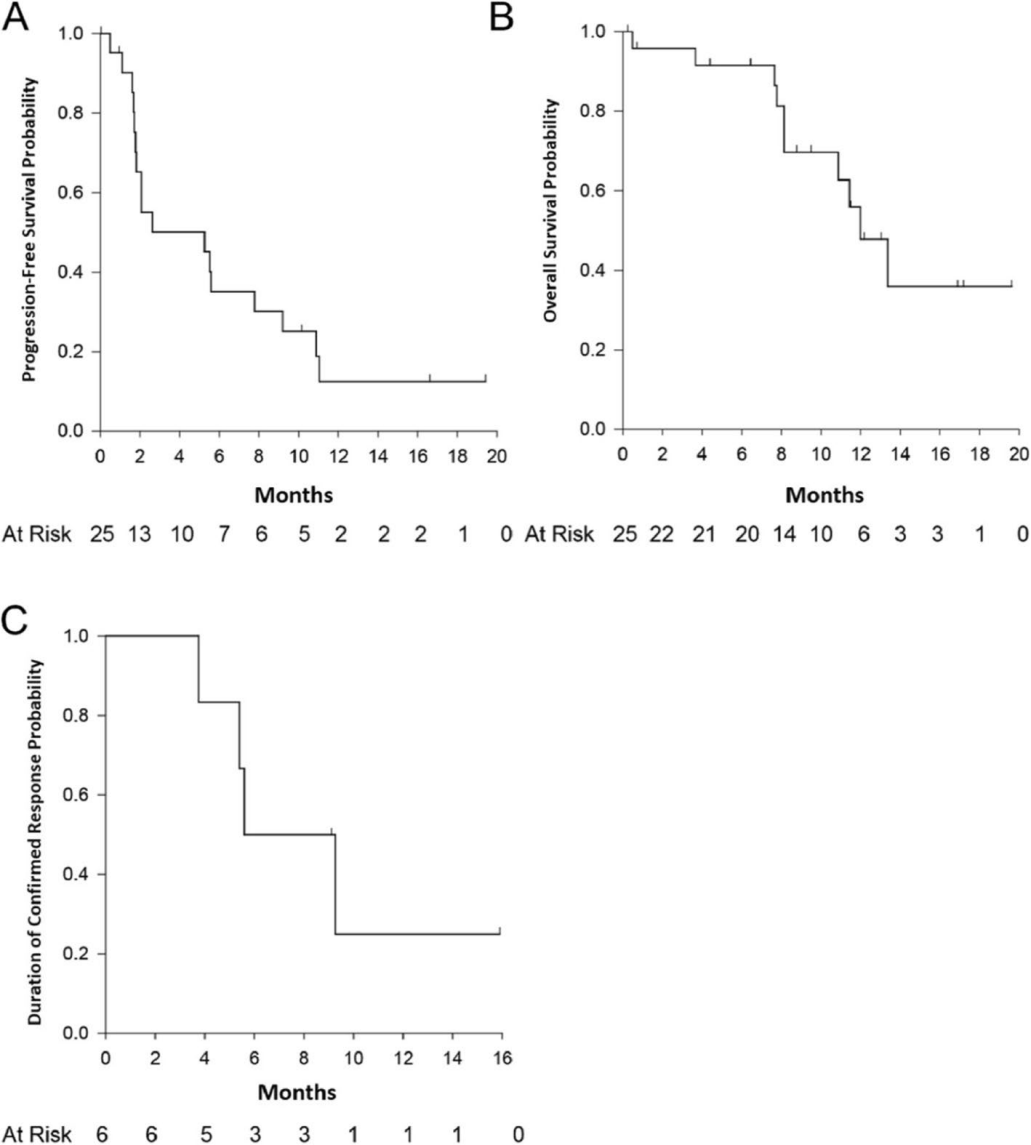
Efficacy outcomes in the phase II NSCLC cohort. Kaplan–Meier plots of Progression-Free Survival (**A**) Overall Survival (**B**) and Duration of Response (**C**)

No patients in the phase Ib portion of the study achieved CR or PR, though stable disease (SD) was observed in seven patients across the four dosing schedules. In the phase II NSCLC portion of the trial, 6 (24%) patients had PR, no patient achieved CR, 4 (16%) had stable disease (SD), 9 (36%) patients had progressive disease (PD), 1 of 9 progressed patients had confirmed PR after initial pseudo-progression (delayed responder), and 6 (24%) patients were deemed not evaluable (NE) due to treatment discontinuation (Fig. 1). Reasons for treatment discontinuation included AEs, subject decision, and PD. The median DOR was 7.41 months (95% CI: 3.75, not reached [NR]), and the median PFS was 5.26 months (95% CI: 1.77, 9.20) (Fig. 2). The median OS was 11.99 months (95% CI: 8.15, NR).

### Pharmacokinetics

Phase Ib PK data showed rapid absorption (1–3 h) and elimination of galunisertib within 48 h. Observed galunisertib plasma concentrations were comparable with those observed in previous galunisertib trials [30].

There was no significant immunogenicity observed that would have impacted study results.

### Biomarkers

Tumor PD-L1 expression was measured by immunohistochemistry. Among 7 responders, including the delayed responder, only 1 patient had high PD-L1 expression (≥ 50%), 5 patients had PD-L1 scores in the range of 1 to 30%, and 1 patient was PD-L1 negative. PD-L1 expression did not correlate with efficacy based upon this small sample set (Fig. 1).

**Fig. 3:**
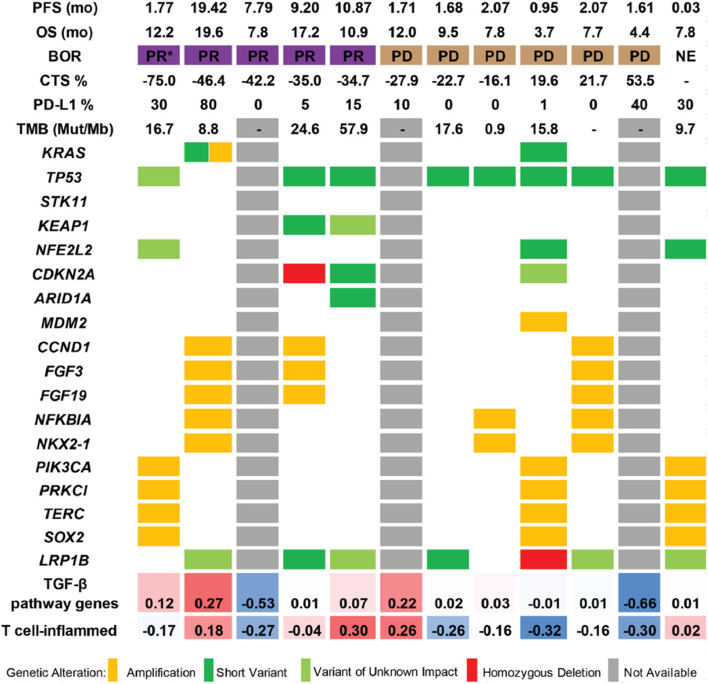
Genetic Variants and RNA Signatures in NSCLC Cohort. Abbreviations: BOR, best overall response; CTS, change in tumor size; NE, not evaluable; OS, overall survival; PD, progressive disease; PD-L1, programmed death ligand 1; PFS, progression-free survival; PR, partial response; TGF-β, transforming growth factor beta; TMB, tumor mutation burden. *pseudo-progressor patient

DNA sequencing was performed for the subset of tumor samples with sufficient tissue remaining (*N* = 9) (Fig. 3). As expected, the *TP53* gene had the greatest frequency of pathogenic variants detected (8 of 9, 89%). Two of the samples associated with PR harbored variants in *CDKN2A* and 2 other samples with *NFE2L2* variants were both associated with PD. No *STK11* mutant tumors were represented in the subset of samples with genetic data, but 2 *KEAP1* mutated tumors were observed in patients with a PR (Fig. 3). This data set was not powered to correlate individual gene alterations or TMB with clinical response.

Baseline RNA expression data associated with 12 tumor samples was used to evaluate previously identified expression signatures associated with immune function and TGF-β biology, including the T-cell inflamed signature and TGF-β pathway genes [31]. Within this small data set elevated expression of TGF-β pathway and T-cell inflamed status tended to be aligned with tumor regression and survival (Fig. 3).

**Fig. 4:**
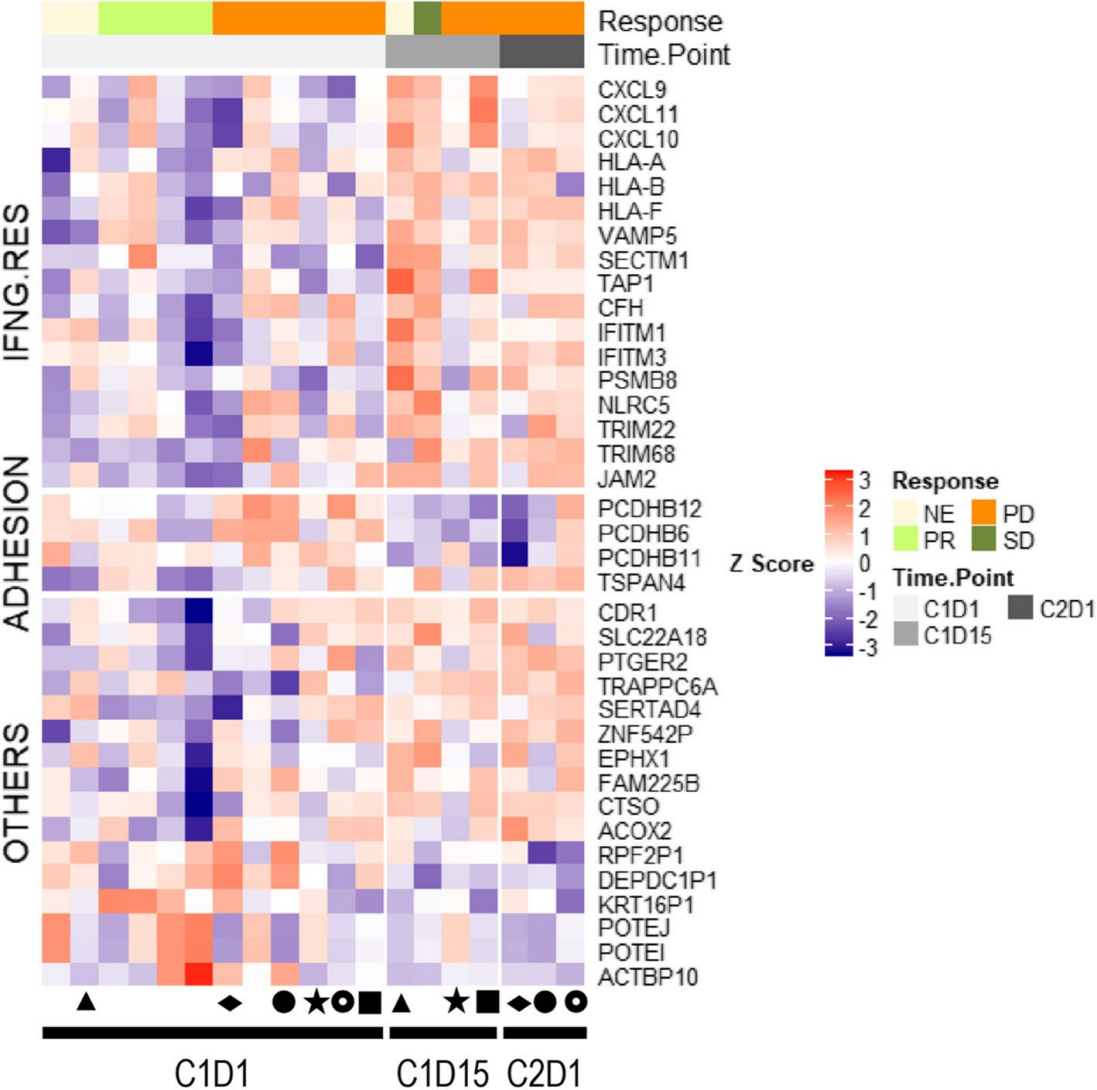
Modulation of Tumor RNA Expression in NSCLC Cohort. Abbreviations: CxDx, Cycle x Day x; FC, fold change; IFNG.RES, interferon gamma response; NE, not evaluable; PD, progressive disease; PR, partial response; SD, stable disease. Shapes represent 6 paired samples; the star represents the pseudo-progressor patient; filtering criteria: *p* ≤ 0.05 and |FC|≥ 1.5

The RNA expression profile of 19 tumor samples were analyzed (12 baseline samples vs 4 collected on C1D15 and 3 on C2D1) to look for pharmacodynamics (treatment driven) expression changes. Of the 6 paired samples, 3 were baseline versus C1D15 and 3 were baseline versus C2D1. Genes associated with interferon gamma response were upregulated, while repressed genes were associated with cell adhesion (Fig. 4). TGF-β pathway genes were not modulated (data not shown). The genes with the largest fold induction at C1D15 were the chemokines *CXCL10, CXCL9,* and *CXC11,* which play a role in attracting immune cells, such as cytotoxic T-cells, natural killer (NK) cells, and macrophages. Only *CXCL9* was induced at C2D1 (unadjusted *p*-value < 0.05). These changes were not associated with response as the paired samples were collected from no-responders.

**Fig. 5:**
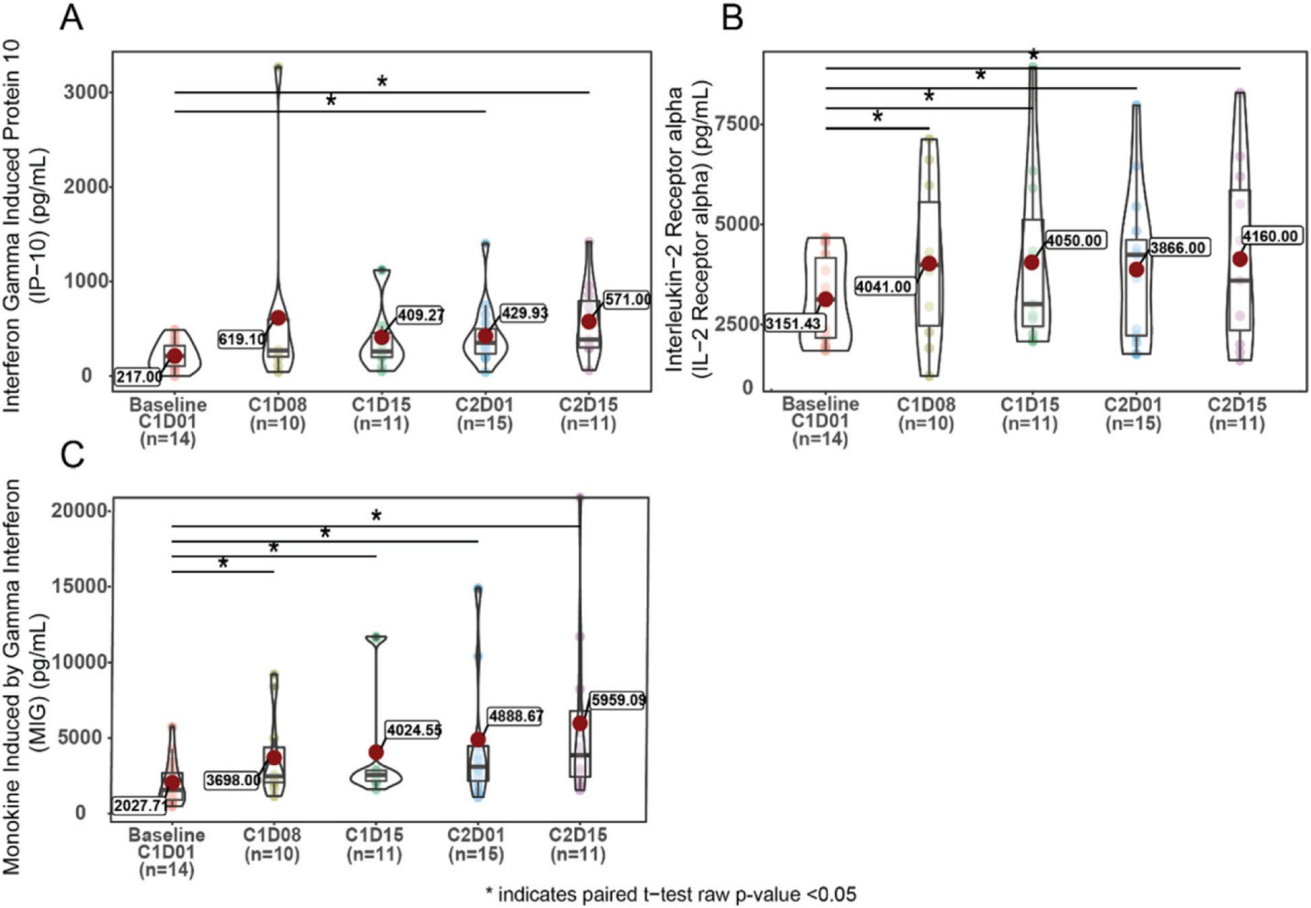
Serum Interferon Gamma Induced Protein 10, Interleukin-2 Receptor Alpha, and Monokine Induced by Gamma Interferon in NSCLC Cohort. Abbreviations: NSCLC, non-small cell lung cancer; CxDx, Cycle X Day X; IP-10, interferon gamma-induced protein 10; MIG, monokine induced by gamma interferon

Serum proteins (*n* = 51) were assessed at baseline and at specific time points during the dosing period. No significant association between baseline protein levels and PFS, OS, or response were detected after adjusting for multiplicity (*n* = 19 patients). Analysis of serum protein modulation in response to treatment (PK), also did not detect changes significant after correction for multiplicity, however, a trend of increased interferon gamma-induced protein 10 (IP-10) (*CXCL10*), monokine induced by gamma interferon (MIG) (*CXCL9*), and interleukin-2 receptor alpha was observed on treatment (Fig. 5). The increase in circulating IP-10 and MIG is consistent with the observed increase in RNA expression of their encoding genes *CXCL10* and *CXCL9* in tumor samples.

## Discussion

Immune checkpoint molecules are established targets for cancer immunotherapies. Blocking PD-(L)1 signaling has become standard of care in patients with lung cancer in different clinical settings, but still not all patients benefit from these therapeutic approaches [32]. Several studies have demonstrated the benefit of anti-PD-(L)1 in combination with chemotherapy and studies are now investigating whether combined blockade of PD-(L)1 and TGF-β signaling can induce tumor regression [33–35]. TGF-β signaling plays an important role in tumorigenesis by inducing EMT. EMT is a major contributing factor of mortality in a range of malignancies including NSCLC, since it is associated with cancer progression and metastasis [13–15, 36].

Overall, in this study we did not observe significant PFS or OS benefit suggesting a delay in disease progression, although some patients that had elevated baseline expression of TGF-β pathway genes tended to have longer PFS and OS benefits than patients who did not (Fig. 3).

Given the immune suppressive role of TGF-β on cytotoxic T-cells, our clinical hypothesis was that dual blockade of both TGF-β and PD-1 may overcome the immunosuppressive nature of the tumor microenvironment and lead to T-cell infiltration and activation [37]. In our study, we observed the combination treatment of galunisertib at the recommended phase II dose of 150 mg BID for 14 days on/14 days off with nivolumab 3 mg/kg every 2 weeks to be well tolerated. No DLTs were observed in the phase Ib portion of the study and the safety profile of the combination was not different than the safety profiles of nivolumab or other checkpoint inhibitors. The most commonly reported treatment-related AEs were pruritus, fatigue, and decreased appetite. Except for 1 myocardial infarction leading to death, deemed unrelated to study treatment due to underlying comorbidities and a familial history of cardiac disease in the patient, no cardiovascular toxicities were observed, which was a major concern from preclinical toxicology studies.

The 2-week treatment break and a maximum dose of 150 mg BID was introduced not to exceed area under the curve (AUC) levels corresponding to levels where valvulopathies were observed in animals. Although we did not observe major cardiovascular signals or emergence of secondary malignancies in our study, it is plausible higher doses might have led to better target inhibition and thus higher response rates. Interestingly, TGF-β pathway genes were not modulated but changes were seen in interferon gamma gene expression that is likely related to PD-1 blockade. Although most patients had tumor target reduction, overall, the efficacy data were not different than PD-(L)1 monotherapy in the same disease setting.

The median OS was 11.99 months (95% CI: 8.15, NR) for patients treated with galunisertib plus nivolumab. This is not different than survival data observed in multiple large studies showing median OS to be between 12–14 month for anti PD-1 (nivolumab, pembrolizumab) and anti PD-L1 (durvalumab, atezolizumab) single agent treatment [4, 38–40]. Here, we report the median PFS to be 5.26 months. This compares favorably to single agent PD-(L)1 trials where median PFS was between 2.8 to 3.8 months depending on the trial [4, 5]. However, given the small sample size and the nonrandomized design of this trial, it is not possible to draw any conclusions as to whether the addition of the TGF-β inhibitor provided any clinical benefit. Studies have shown variable associations between PD-L1 expression and response to nivolumab in NSCLC [4, 41–43]. Here, we did not observe clear associations between response and PD-L1 expression due to the limited sample size, however most patients with PD-L1 positivity had tumor reduction (Fig. 3).

DNA sequencing was performed to assess for associations between genetic variants and clinical response. Both *STK11* and *KEAP1* mutations have been associated with resistance to checkpoint blockade, especially when concurrent with kirsten rat sarcoma viral oncogene homolog (*KRAS)* mutations [44, 45]. None of the patients that had tumor tissue available for sequencing had *STK11* mutations suggesting loss of liver kinase B1. Two patients harbored a *KEAP1* mutation, which has been associated with tumor progression and treatment resistance in lung cancer [46, 47]. Interestingly, both patients with a *KEAP1* mutation achieved a PR, despite the association with poor response to checkpoint inhibitors in combination with chemotherapy in first line therapy [45]. Two samples had a *KRAS* mutation. One *KRAS (G12C)* variant without co-occurring mutations in *TP53* and *STK11* achieved a PR, while the other with *KRAS (G12S)* variant concurrent with a *TP53* mutation progressed on treatment. An *ARID1A* mutation was found in 1 patient sample, which has been associated with a better response to checkpoint inhibitors and the patient response observed here was a PR [48, 49]. Finally, TMB is a surrogate of tumor antigenicity and has been positively associated with a response to nivolumab and other checkpoint inhibitors [12, 50, 51]. In our study, no association of TMB score with response was observed, however, data are insufficient to make any conclusions.

Patients were not selected prospectively based on TGF-β gene expression, therefore the appropriate patient population may not have been treated to see changes in the expression of TGF-β pathway genes. Higher doses may have modulated gene expression but were associated with higher risk of inducing cardiac toxicity. Notwithstanding, we detected increased transcription and serum levels of T-cell activation and migration markers IP-10 (*CXCL10*) and MIG (*CXCL9*) following combination treatment of nivolumab and galunisertib. Similarly, increased transcription and serum levels of these same markers were reported in renal cell carcinoma patients post nivolumab treatment [52]. These changes may be indicative of treatment driven T-cell recruitment to the tumor microenvironment.

These findings demonstrate combination treatment with galunisertib plus nivolumab has an acceptable safety profile in patients with refractory or recurrent NSCLC who received prior platinum-based treatment and were treatment-naive for anti-PD-(L)1 or TGF-β receptor 1 kinase inhibitors. Notwithstanding, this study has several limiting factors. The primary limitation is the low number of patients. While this study contributes to research in the field, analysis of a larger cohort of patients would provide further data on the safety and efficacy of the combination treatment in NSCLC. Another limitation is the low number of paired baseline and on-treatment tumor samples for exploratory comparative analysis. Collection of baseline and on-treatment biopsies were required for participation in this study, however, limited on-treatment tissue was submitted due to tumor progression, clinical deterioration, insufficient tumor, and patient decision. Thus, although these results identify potential predictive markers of therapeutic effect in recurrent or refractory NSCLC, more patient samples are needed to support the translational work.

In conclusion, combination treatment of galunisertib with nivolumab was well tolerated and the study met its primary endpoint of safety. Preliminary efficacy activity was observed in a subset of patients and was not associated with tumor PD-L1 expression. Increased levels of T-cell activation and migration markers were indicative of potential treatment-driven T-cell recruitment. However, the sample size was small and the magnitude of response or benefit in survival outcomes do not clearly differentiate from nivolumab monotherapy in the second line IO-naïve setting. In this unselected cohort of NSCLC patients, a few patients exhibited a baseline increase in TGF-β activity that may have benefited from this combination treatment. Further studies that select patients based on TGF-β pathway expression may yield higher response rates to TGF-β and immune checkpoint inhibitor combination treatment.

## Supplementary Information

**Additional file 1.** Trial inclusion and exclusion criteria.

**Additional file 2.** Phase Ib baseline patient demographics.

## Data Availability

The data that support the findings of this study are available from Eli Lilly and Company but restrictions apply to the availability of these data, which were used under license for the current study, and so are not publicly available. Data are however available from the authors (Emin Avsar, Ivelina Gueorguieva, Michael Man, Shawn T Estrem, Jiangang Liu) upon reasonable request and with permission of Eli Lilly and Company.

## Abbreviations

AE: Adverse event
ARID1A: AT-rich interaction domain 1A
AUC: Area under the curve
BID: Twice daily
BOR: Best overall response
CDKN2A: Cyclin dependent kinase inhibitor 2A
CI: Confidence interval
CR: Complete response
CTCAE: Common Terminology Criteria for Adverse Events
CTS: Change in tumor size
CXCL: C-X-C motif chemokine ligand
CxDy: Cycle x Day y
DLT: Dose-limiting toxicity
DOR: Duration of response
ECOG: Eastern Cooperative Oncology Group
EMT: Epithelial-mesenchymal transition
IP-10: Interferon gamma-induced protein 10
KEAP1: Kelch-like ECH-associated protein 1
KRAS: Kirsten rat sarcoma viral oncogene homolog
MIG: Monokine induced by gamma interferon
NE: Not evaluable
NFE2L2: Nuclear factor, erythroid 2 like 2
NK: Natural killer
NR: Not reached
NSCLC: Non-small cell lung cancer
OS: Overall survival
PD: Progressive disease
PD-1: Programmed cell-death-1
PD-L1: PD-1 ligand
PFS: Progression-free survival
PK: Pharmacokinetics
PR: Partial response
RECIST: Response evaluation criteria in solid tumors
RNA: Ribonucleic acid
SD: Stable disease
SMAD2/3/4: Mothers against decapentaplegic homolog 2/3/4
STK11: Serine/threonine kinase 11
TGF-β: Transforming growth factor β
TKI: Tyrosine kinase inhibitor
TMB: Tumor mutation burden
TP53 gene: Tumor protein p53
