# Supplementary material for: A phase Ib/II study of galunisertib in combination with nivolumab in solid tumors and non-small cell lung cancer
Source: BMC Cancer. 2023 Jul 28;23:708. doi: 10.1186/s12885-023-11153-1 (PMC10386782; doi:10.1186/s12885-023-11153-1)
Supplement: Supplementary file 1 — Additional file 1. Trial inclusion and exclusion criteria. [file 12885_2023_11153_MOESM1_ESM.docx]

**Additional File 1: Trial Inclusion and Exclusion Criteria.**

|  | **Inclusion criteria** | **Exclusion criteria** |
| --- | --- | --- |
| **Overall study criteria** | Histological or cytological confirmation of disease | Have received treatment within 28 days or 5 half-lives of the initial dose of study drug with an investigational product or nonapproved use of a drug or device for noncancer indications or are concurrently enrolled in any other type of medical research judged not to be scientifically or medically  compatible with this study. |
|  | For glioma, previous or current histologically confirmed diagnosis of World Health Organization Grade IV malignant glioma; and first recurrence of glioma as determined by RANO criteria following standard chemoradiation. If first recurrence of glioma is documented by MRI, an interval of at least 12 weeks after the end of prior radiation therapy is required unless there is either: i) histopathologic confirmation of recurrent tumour, or ii) new enhancement on MRI outside of the radiotherapy treatment field. For glioma patients, biopsy confirmation of recurrent disease is not required as long as new enhancement on MRI outside of the radiotherapy treatment field is documented. | Have moderate or severe cardiovascular disease |
|  | For glioma, have measurable disease as defined by RANO for the first 25 patients. If the cohort is expanded per the Simon 2-stage design, then patients with either measurable or non-measurable disease as defined by RANO are eligible. If measurable disease is present, at least 1 measurable glioma lesion present prior to randomization must meet the following criteria: contrast enhancing and clearly defined, bi-dimensionally measurable margins and at least 2 perpendicular diameters measuring ≥10 mm x ≥10 mm (Note: MRI measurements will not include surgical cavities, cysts, or necrotic area) |  |
|  | For non-neuro-oncology tumours, have measurable disease as defined by: RECIST version 1.1 | Have a serious concomitant systemic disorder including active infection with HIV (HIV 1/2 antibodies). |
|  | For patients other than glioma patients, baseline tumour biopsies are required for all cohorts. For glioma patients, have new biopsy or archived tumour tissue available | Active infection with HBV; HCV is allowed only in HCC patients. HCC patients at risk for HBV reactivation are only eligible in the HCC cohort |
|  | ≥18 years of age | Are receiving immunosuppressive agents (eg, cyclosporine) for any reason except for corticosteroids for transfusions. Low-dose corticosteroids for glioma are permitted |
|  | Must have recovered from the toxicity and or complications from any recent surgery | Have had prior monoclonal antibody therapy within 28 days before Study Day 1 or not recovered (≤ Grade 1 or at baseline) from AEs (except for fatigue or alopecia) due to agents administered more than 28 days earlier |
|  | Given written informed consent | Have had prior therapy with an anti–PD-1, anti–PD-L1, anti–PD-L2, anti-CD137, or anti–cytotoxic T lymphocyte-associated antigen-4 antibody (including ipilimumab or any other antibody or drug specifically targeting T-cell co-stimulation or checkpoint pathways) or a TGFβ R1 kinase inhibitor |
|  | ECOG PS of 0 or 1 | Have a second concurrent primary malignancy that, in the judgment of the investigator and Lilly, may affect the interpretation of results |
|  | Have an INR or prothrombin time ≤1.5xULN, except for HCC, unless patient is receiving stable dose anticoagulant therapy | Have prior malignancies. Patients with carcinoma in situ of any origin and patients with prior malignancies who are in remission and whose likelihood of recurrence is very low (such as basal cell carcinoma), as judged by the Lilly clinical research physician, are eligible for this study. |
|  | Discontinued previous cancer treatments for at least 28 days and recovered from the acute effects of therapy and at least 42 days for mitomycin-C or nitrosoureas | Receiving any concurrent antineoplastic therapy |
|  | Reliable, available for duration of study, and willing to follow study procedures | Have had prior radiotherapy within 2 weeks of therapy. Patients must have  recovered from all radiation-related toxicities, not require more than low-dose corticosteroids (except for low-dose corticosteroids for glioma, eg, prednisone 10 mg or less daily or equivalent dose of other corticosteroid), and not have had radiation pneumonitis. A one-week washout is permitted for palliative  radiation to non-CNS disease with Lilly physician approval. |
|  | Use an approved contraceptive method during the study and for 23 weeks for women of childbearing potential and 31 weeks for males sexually active with women of childbearing potential after discontinuation of study treatment. | For glioma patients, have diffuse leptomeningeal disease |
|  | Women of childbearing potential must have a negative β-human chorionic  gonadotropin pregnancy test documented within 7 days prior to treatment. Should a woman become pregnant or suspect she is pregnant while participating in this study, she should immediately discontinue study treatment and inform her treating physician immediately. If pregnancy is confirmed, patient will be discontinued from treatment. | For non-glioma patients, patients with known active CNS metastases and/or carcinomatous meningitis. Patients with stable, previously treated brain metastases may participate if neurologic symptoms have resolved, they have been off steroids for at least 7 days, and have no evidence of disease progression by imaging for at least 2 weeks before the first dose of study treatment. |
|  | Have an estimated life expectancy of ≥12 weeks | Have active known or suspected autoimmune disease or a documented history of autoimmune disease, or a syndrome that requires systemic steroids or immunosuppressive agents. Patients with vitiligo, alopecia, type I diabetes mellitus, residual hypothyroidism due to autoimmune condition only requiring thyroid replacement therapy (must be on a stable dose started prior to study  entry), and/or psoriasis not requiring chronic and systemic  immunosuppressive treatment, are permitted to enrol if the above listed  conditions are not expected to recur in the absence of an external trigger. |
|  | Are able to swallow tablets | Patients requiring escalating or chronic supraphysiologic doses of corticosteroids for control of their disease at randomization are excluded. However, patients with resolved childhood asthma/atopy, who require intermittent use of bronchodilators or local steroid injections, who have hypothyroidism stable on thyroid replacement therapy at study entry, or who have Sjörgen's syndrome are eligible for the study. |
|  |  | Patients with Crohn’s disease are not eligible |
|  |  | Concurrent treatment with Novo-Tumor Treating Fields® (Novocure; Haifa, Israel) is not permitted. Prior whole brain or gamma knife radiosurgery is  permitted for the treatment of glioma or metastatic brain lesions. |
|  |  | Have evidence of interstitial lung disease that is symptomatic or may interfere with the detection or management of suspected drug-related pulmonary toxicity or active, non-infectious pneumonitis |
|  |  | Have an active infection requiring systemic therapy |
|  |  | Be pregnant or nursing |
|  |  | Have received a live vaccine within 30 days before the first dose of study treatment |
|  |  | Have a GI condition that may affect drug absorption |
|  |  | Have a history or current evidence of any condition, therapy, or laboratory  abnormality that might confound the results of the study, interfere with the  patient's participation for the full duration of the study, or is not in the best interest of the patient to participate, in the opinion of the treating investigator. |
|  |  | Have known psychiatric or substance abuse disorders that in the opinion of the investigator would interfere with cooperation with the requirements of the study. |
|  |  | Have a history of allergy or hypersensitivity to study drug components |
|  |  | For HCC, have known HCC with fibro-lamellar or mixed histology |
|  |  | For HCC, have presence of clinically relevant ascites |
|  |  | For HCC, have a history of hepatic encephalopathy |
|  |  | For HCC, have had a liver transplant |
|  |  | For HCC, have experienced any CTCAE Grade 3 or 4 GI bleeding or any variceal bleeding episode in the 3 months prior to enrolment requiring transfusion or endoscopic or operative intervention. (Patients with any bleeding episode considered life-threatening during the 3 months prior to enrolment are excluded, regardless of transfusion or intervention status.) |
|  |  | For HCC: have oesophageal or gastric varices that require immediate intervention (eg, banding, sclerotherapy) or represent a high bleeding risk in the opinion of the investigator or consulting gastroenterologist or hepatologist |
|  |  | For glioma patients only, have diffuse leptomeningeal disease |
|  |  | For glioma patients only, previous treatment with carmustine wafer except when administered as first-line treatment and at least 6 months prior to randomization |
| **Phase Ib specific criteria** | Advanced refractory solid tumours in any line of treatment. |  |
|  | Sufficient haematological function (platelet count of ≥100x10^9^/L, haemoglobin concentration ≥9.0 g/dL or ≥5.6 mmol/L, and ANC≥1.5x10^9^/L) |  |
|  | Sufficient hepatic function (bilirubin ≤1.5xULN) and ALT and AST levels ≤3.0xULN |  |
|  | Serum creatinine ≤1.5 ULN × institutional ULN OR measured or calculated CrCl ≥50 mL/min for patients with creatinine levels ≤1.5x institutional ULN. |  |
| **Phase II specific criteria** | Recurrent or refractory NSCLC, HCC with AFP ≥200 ng/mL, or glioma (primary) | ≥1 prior line of therapy |
|  | Patients must agree to undergo an on-study biopsy |  |
|  | Have had disease progression or be refractory or intolerant to 1 prior line of therapy (first-line therapy) for recurrent or refractory for NSCLC or HCC and have refused currently approved second-line of therapy. First-line therapy may include multiple chemotherapeutic, targeted or immunotherapeutic agents with or without radiation therapy and/or surgery. Each subsequent line of therapy is preceded by disease progression. A switch of an agent within the same drug class within a regimen in order to manage toxicity does not define the start of a new line of therapy. |  |
|  | For NSCLC: Prior lines of therapy must include a platinum-based therapy. Investigational agents used in combination with standard therapies are allowed. Patients who received platinum-based neoadjuvant or adjuvant therapy and subsequently received platinum-based therapy as first-line of therapy are eligible. |  |
|  | For NSCLC: Patients who have completed neoadjuvant or adjuvant therapy with a platinum doublet and have experienced disease recurrence within 6 months of completing the platinum doublet are eligible |  |
|  | For NSCLC: Tumours with driver mutations (epidermal growth factor receptor mutation positive or anaplastic lymphoma kinase fusion oncogene positive) treated with a tyrosine kinase inhibitor or crizotinib are eligible. For patients who have progressed on a tyrosine kinase inhibitor or crizotinib or are intolerant to this targeted therapy, that patient must receive platinum-based therapy prior to enrolment in this study. Documentation of such mutations must be available and entered into the electronic case report form. |  |
|  | For NSCLC: Maintenance or switch maintenance therapy after first-line chemotherapy will be considered part of the first-line regimen and is acceptable. |  |
|  | For NSCLC: Patients who completed and progressed on a platinum-containing regimen as adjuvant, neoadjuvant, or part of a course of chemoradiation therapy given from locally advanced disease and developed recurrent (local or metastatic) disease within the 6 months before screening would be counted as having received 1 prior platinum-containing regimen and therefore would not require retreatment with a platinum-containing regimen for Stage IIIB, IV, or recurrent disease and are eligible. However, patients must have received at least 2 cycles of platinum doublet-based chemotherapy before discontinuation for toxicity. If patients received only 1 cycle of a platinum doublet and discontinue due to clear progression, that regimen should be counted as a prior line of therapy. |  |
|  | For HCC: One prior line of therapy which must include sorafenib, or patient must have progressed or been intolerant to sorafenib for patients not eligible for trans-arterial chemoembolization. Patients who had sorafenib for locally advanced disease or are intolerant to sorafenib are eligible. Patients may have had clinical progression only following sorafenib or local therapy. |  |
|  | For HCC: Must have Child-Pugh A only. Patients may have any viral status (hepatitis B, hepatitis C, or none). |  |
|  | For HCC: have a viral load <100 IU/mL |  |
|  | For HCC: For hepatitis B patients, must be on a nucleoside analogue reverse transcriptase inhibitor (lamivudine, telbivudine, adefovir, tenofovir, or entecavir) |  |
|  | For Glioma: Previous first-line of therapy with at least radiotherapy and temozolomide |  |
|  | Serum creatinine ≤1.5 ULN × institutional ULN OR measured or calculated CrCl (glomerular filtration rate can also be used in place of creatinine or CrCl) ≥50 mL/min for patients with creatinine levels ≤1.5x institutional ULN. Patients with ≤ Grade 2 neuropathy are eligible and may enrol |  |
|  | For NSCLC cohort only: Haematologic: platelets ≥100x10^9^/L, and haemoglobin ≥9 g/dL or ≥5.6 mmol/L, ANC ≥1.5x10^9^/L. Patients may receive erythrocyte transfusions to achieve this haemoglobin level at the discretion of the investigator (at least 3 days before starting treatment with study drug). Initial treatment must not begin until 2 days after the erythrocyte transfusion and after the confirmation of haemoglobin level ≥9 g/dL. Have sufficient hepatic function, defined as bilirubin ≤1.5xULN (except patients with Gilbert Syndrome who must have a total bilirubin level of <3.0xULN) and ALT and AST levels ≤3.0xULN. |  |
|  | For HCC cohort only: Haematologic: platelets ≥50 x 10^9^/L, haemoglobin ≥9 g/dL or ≥5.6 mmol/L, ANC ≥1.5x10^9^/L. Patients may receive erythrocyte transfusions to achieve this haemoglobin level at the discretion of the investigator (at least 3 days before starting treatment with study drug). Initial treatment must not begin until 2 days after the erythrocyte transfusion and after the confirmation of haemoglobin level ≥9 g/dL. Hepatic: bilirubin ≤2.5xULN (except patients with Gilbert Syndrome who must have a total bilirubin level of <3.0xULN), ALT, and AST ≤5.0xULN. Prothrombin time/ INR ≤2.3; or prothrombin time 6 seconds above control |  |

AE = adverse event, AFP = α-foetoprotein, ANC = Absolute neutrophil count, CNS = central nervous system, CTCAE = Common Terminology Criteria for Adverse Events , ECOG = Eastern Cooperative Oncology Group Performance Score, GI = Gastrointestinal, HCC = hepatocellular carcinoma, HIV = human immunodeficiency virus, HBV = hepatitis B virus, HCV = hepatitis C virus, INR = international normalized ratio, MRI = magnetic resonance imaging, NSCLC = non-small cell lung cancer, PD-1 = programmed cell death–1, PD-L1 = programmed cell death–ligand 1, PD-L2 = programmed cell death–ligand 2, RECIST = Response Evaluation Criteria in Solid Tumors, TGF-β = transforming growth factor-β, ULN = upper limit of normal
