# Supplementary material for: A phase Ib/II study of galunisertib in combination with nivolumab in solid tumors and non-small cell lung cancer
Source: BMC Cancer. 2023 Jul 28;23:708. doi: 10.1186/s12885-023-11153-1 (PMC10386782; doi:10.1186/s12885-023-11153-1)
Supplement: Supplementary file 2 — Additional file 2. Phase Ib baseline patient demographics. [file 12885_2023_11153_MOESM2_ESM.docx]

**Additional File 2: Phase Ib Baseline Patient Demographics**

| **Characteristics** | **Galunisertib 50 mg QD (N=3)**  **n (%)** | **Galunisertib 50 mg BID (N=5)**  **n (%)** | **Galunisertib 80 mg BID (N=3)**  **n (%)** | **Galunisertib 150 mg BID (N=4)**  **n (%)** |
| --- | --- | --- | --- | --- |
| **Pathological diagnosis** |  |  |  |  |
| AC of the distal esophagus | 1 (33.3) | 0 (0.0) | 0 (0.0) | 0 (0.0) |
| Anaplastic TC | 0 (0.0) | 1 (20.0) | 0 (0.0) | 0 (0.0) |
| Appendiceal AC | 0 (0.0) | 0 (0.0) | 1 (33.3) | 1 (25.0) |
| Clear cell carcinoma of Mullerian origin | 0 (0.0) | 0 (0.0) | 0 (0.0) | 1 (25.0) |
| Ewing’s sarcoma of pelvis | 0 (0.0) | 1 (20.0) | 0 (0.0) | 0 (0.0) |
| Ewing’s sarcoma | 0 (0.0) | 1 (20.0) | 0 (0.0) | 0 (0.0) |
| Glioblastoma | 0 (0.0) | 1 (20.0) | 1 (33.3) | 1 (25.0) |
| HCC | 0 (0.0) | 1 (20.0) | 0 (0.0) | 0 (0.0) |
| Leiomyosarcoma with osteosarcomatous differentiation | 1 (33.3) | 0 (0.0) | 0 (0.0) | 0 (0.0) |
| Infiltrating Mammary AC | 1 (33.3) | 0 (0.0) | 1 (33.3) | 0 (0.0) |
| Malignant neoplasm of uterus | 0 (0.0) | 0 (0.0) | 0 (0.0) | 1 (25.0) |
| **Sex^a^** |  |  |  |  |
| Female | 1 (33.3) | 1 (20.0) | 1 (33.3) | 3 (75.0) |
| Male | 2 (66.7) | 4 (80.0) | 2 (66.7) | 1 (25.0) |
| **Age, median (range)** | 63 (44-75) | 50 (27-63) | 34 (32-42) | 64 (49-69) |
| **Race^a^** |  |  |  |  |
| Black or African | 1 (33.3) | 0 (0.0) | 0 (0.0) | 1 (25.0) |
| White | 2 (66.7) | 5 (100.0) | 3 (100.0) | 3 (75.0) |
| **Ethnicity^a^** |  |  |  |  |
| Not Hispanic or Latino | 3 (100.0) | 3 (100.0) | 2 (100.0) | 4 (100.0) |
| **Baseline ECOG performance status** |  |  |  |  |
| 0 | 1 (33.3) | 1 (20.0) | 1 (33.3) | 2 (50.0) |
| 1 | 2 (66.7) | 3 (60.0) | 2 (66.7) | 2 (50.0) |
| **Tobacco use** |  |  |  |  |
| Current | 0 (0.0) | 3 (60.0) | 0 (0.0) | 0 (0.0) |
| Former | 2 (66.7) | 0 (0.0) | 3 (100.0) | 1 (25.0) |
| Never | 1 (33.3) | 2 (40.0) | 0 (0.0) | 3 (75.0) |
| **Prior therapy** |  |  |  |  |
| 1 prior regimen | 0 (0.0) | 0 (0.0) | 0 (0.0) | 3 (75.0) |
| 2 prior regimens | 0 (0.0) | 1 (20.0) | 1 (33.3) | 0 (0.0) |
| ≥3 prior regimens | 3 (100.0) | 4 (80.0) | 2 (66.7) | 1 (25.0) |

ECOG PS = Eastern Cooperative Oncology Group performance status, NSCLC = non-small cell lung cancer, N = number of subjects in the safety population, n = number of patients in the specific category, QD = once daily, BID = twice daily, AC = adenocarcinoma, BC = breast cancer, HCC = hepatocellular carcinoma, TC = thyroid cancer

^a^The number of subjects with non-missing data were used as denominator
